# The role of dermocosmetics in the management of cancer-related skin toxicities: international expert consensus

**DOI:** 10.1007/s00520-023-08116-4

**Published:** 2023-11-04

**Authors:** Brigitte Dreno, Kiarash Khosrotehrani, Giselle De Barros Silva, Julie Ryan Wolf, Delphine Kerob, Mark Trombetta, Etienne Atenguena, Pascale Dielenseger, Meng Pan, Florian Scotte, Ivan Krakowski, Mario Lacouture

**Affiliations:** 1 Nantes Université, INSERM, CNRS, Immunology and New Concepts in Immunotherapy, INCIT, UMR 1302/EMR6001. F-44000, Nantes, France; 2 Experimental Dermatology Group, University of Queensland Diamantina Institute, Brisbane, Australia; 3 Department of Oncology, Hospital Alemao Oswaldo Cruz, Sao Paulo, Brazil; 4 Departments of Dermatology and Radiation Oncology, School of Medicine and Dentistry, University of Rochester Medical Center, Rochester, NY USA; 5 La Roche Posay International, Levallois, France; 6 Department of the Radiologic Sciences, Allegheny Health Network, Drexel University College of Medicine, Pittsburgh, PA USA; 7 Department of Internal Medicine, University of Yaoundé I, Yaoundé Hospital General, Yaoundé, Cameroon; 8 Research and Education for Paramedic Professionals Nursing Coordinator, Gustave Roussy, Villejuif, France; 9 École Des Sciences du Cancer, Université Paris Sud (XI), Paris, France; 10 Association Française Des Infirmières de Cancerologie (AFIC), Paris, France; 11 Department of Dermatology, Rui Jin Hospital, Shanghai Jiao Tong University School of Medicine, Shanghai, China; 12 Interdisciplinary Patient Pathway Division, Gustave Roussy, Villejuif, France; 13 Multinational Association of Supportive Care in Cancer (MASCC), Aurora, Canada; 14 Medical Oncologist, Bordeaux, France; 15 Association Francophone Des Soins Oncologiques de Support (AFSOS), Bègles, France; 16 Dermatology Service, Department of Medicine, Memorial Sloan-Kettering Cancer Center, New York, NY USA

**Keywords:** Oncodermatology, Dermocosmetics, Cosmeceuticals, Skin toxicity, Cancer management, Anticancer therapeutics

## Abstract

Skin toxicities are very common in patients undergoing cancer treatment and have been found to occur with all types of cancer therapeutic interventions (cytotoxic chemotherapy, targeted therapies, immunotherapy, and radiotherapy). Further, skin toxicities can lead to interruption or even discontinuation of anticancer treatment in some patients, translating to suboptimal outcomes. Dermocosmetics (or cosmeceuticals)—defined as skincare solutions incorporating dermatologically active ingredients (beyond vehicle effects) that directly improve symptoms of various skin conditions—are increasingly being used in cancer care to prevent and manage skin toxicities. The active ingredients in these products have a measurable biological action in skin; they typically improve skin integrity (barrier function/hydration and other factors) while relieving skin symptoms. The Association Francophone des Soins Oncologiques de Support (AFSOS) and Multinational Association of Supportive Care in Cancer (MASCC) partnered to select a multidisciplinary group of healthcare professionals involved in the management of patients with cancer and skin toxicities. The group reviewed existing literature and created a summary of recommendations for managing these toxicities through online meetings and communication. In this publication, the group (1) reviews new skin toxicities seen with oncology drugs and (2) evaluates the role of dermocosmetics in improving patient outcomes and minimizing cancer treatment interruptions. We provide general recommendations for initiation and selection of skin care in all oncology patients as well as recommendations for what factors should be considered when using dermocosmetics in specific types of skin toxicities.

## Introduction

Recent advances in both anticancer treatments and understanding of pathogenesis of various cancers have led to individualized patient care. This has increased tolerability of treatments and steadily improved outcomes, with major benefits apparent in both the duration of survival and proportion of patients who survive [1–3]. Yet conventional cytotoxic, radiotherapeutic cancer treatments, and newer targeted therapies and immunotherapies have associated adverse events, among which skin toxicities are the most common [2–4]. It is important for members of the cancer treatment team to have solid knowledge of therapy-induced skin toxicities so that management can be optimized [3].

Patients with cancer have unique dermatologic needs, since skin toxicities are prevalent and are associated with a high physical burden [5, 6]. These toxicities may include pruritus, xerosis, facial papules and pustules (drug-induced folliculitis, also known as acneiform rash), hand-foot reaction, alopecia, and other skin problems [5]. They can occur because anticancer agents affect rapidly proliferating cells, which include cancer cells but also normal cells such as skin [7–10]. While skin problems are very common, they can often be given a low priority compared to the clinical tumor responses or life-threatening side effects such as neutropenia [5]. However, skin problems may lead to an impairment in interpersonal and emotional well-being [5]. Further, skin problems may be painful or disfiguring or have a high emotional impact because patients may be forced to reveal their cancer whether they want to or not [1, 4, 5, 11]. Finally, skin problems can affect the patient’s ability to continue anticancer treatment [5, 12]. Several studies have shown that facial skin rashes are associated with a high rate of oncologic treatment dose reductions and treatment discontinuation, which may be detrimental to treatment outcome [8, 12–14]. Patients have indicated that they need more information and support for handling adverse events, including skin toxicities [15].

Researchers are still working to elucidate the exact mechanisms of skin toxicities during cancer therapy [13]. However, it is thought that the primary mechanisms include alterations in skin barrier function and microbiota, inducing mainly inflammation, autoimmune responses, and phototoxicity. The risk for skin toxicities could be increased in older adults and those who are treated with immune checkpoint inhibitors; similarly, pigmentation abnormalities are a risk among those with darker skin types [16]. Minimizing alterations in skin barrier function and photoprotection are key to prevention of skin toxicities.

Since 2006, there has been interest in including dermatologists in the cancer care team, since dermatologic adverse events were frequently responsible for dose modification or interruption of anticancer therapy [17]. Since then the field of oncodermatology has blossomed, and there is increasing access for the cancer care team to dermatology protocols. As recently as 2022, Barrios et al. showed that involvement of dermatologists with the oncology care team increased the rate of positive outcomes and decreased potential for skin toxicity recurrence [18]. These researchers concluded that the result was “impactful reductions in interruption of anticancer therapy” [18]. In 2020, Chen et al. had reported similar beneficial results among a group of patients treated with immune checkpoint inhibitors [19]. The authors came together as a multidisciplinary group of healthcare professionals involved in cancer care—oncologists, radiation therapists, dermatologists, and nurses—with the goal of providing guidance on how to incorporate skin care into cancer management strategies. Today, “skin care” as an umbrella term covers a myriad of products, including a variety of cleansers, moisturizers, emollients, and others. A combination of cleanser, moisturizer, and sunscreen is important for all patients undergoing cancer therapy; in addition, daily skincare with more active ingredients may be prescribed for patients. In recent years, a category of products known as dermocosmetics or cosmeceuticals has risen to prominence [20]. These products can be defined as skincare solutions incorporating dermatologically active ingredients that directly improve symptoms of various skin conditions (beyond any expected vehicle effects). The active ingredients in these products have a measurable biological action in skin; they typically improve skin integrity (barrier function, hydration, and other factors) while relieving skin symptoms. We have provided recommendations for skin care in cancer patients, with an emphasis on dermocosmetics when appropriate.

## Methods

A partnership between Association Francophone des Soins Oncologiques de Support (French-speaking Association for Oncological Supportive Care, AFSOS) and Multinational Association of Supportive Care in Cancer (MASCC) was formed to select a multidisciplinary panel of healthcare professionals involved in the management of patients with cancer and skin toxicities. A total of 10 healthcare professionals represented the fields of dermatology, oncology, radiation sciences, and nursing and came from the main regions of the world.

The group evaluated a literature review of the latest data regarding new drugs approved and side effects as well as studies related to oncology skin supportive care. This review included guidelines, consensus papers, reviews on the management of skin toxicities, as well clinical and other research studies, with a focus on those published in the English language. Experts met for an online 4-h meeting to discuss role of dermocosmetics and their current best practices. The results are summarized in this publication.

## Recommendations

### Skincare in prevention and treatment

**Fig. 1:**
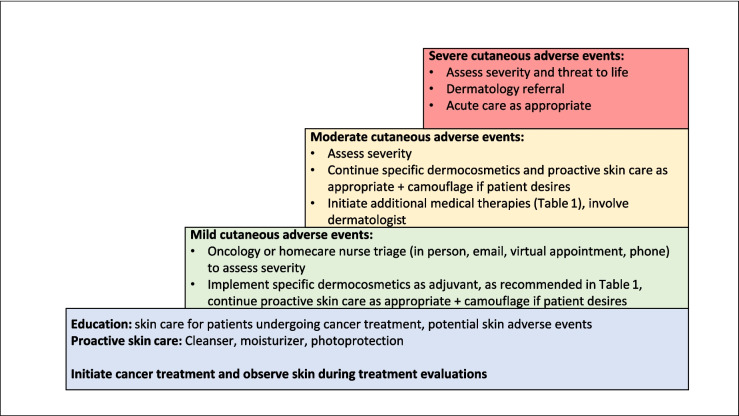
Steps in managing skin toxicities

While management approaches may be individualized in accordance with the specific oncologic treatment and patient risk profile, there are general daily skin care approaches that should be used for the majority of cancer patients from the initiation of therapy [1, 4]. Prevention is a key element of managing skin toxicities, although it is perhaps not as widely used as we could hope [14]. We recommend that the oncology multidisciplinary team familiarize themselves with skin care basics and develop a standard protocol for implementing cleansers and moisturizers for cancer patients, along with sunscreen in the appropriate times of year/regions. Some dermocosmetics are formulated for—and tested in—skin that is fragile, pathological, and sensitive; formulations should be free of additives, irritants such as fragrances or perfumes, sensitizing agents, and herbal extracts (as much as possible) [1, 13]. These may be more appropriate for prevention and management of skin toxicities [13]. Fig. 1 presents a graphic illustration of steps in managing skin toxicities.

Appropriate skin care supports and maintains the epidermal skin barrier and skin microbiota. It is crucial to maintain the diversity of microbiota since this affects innate immunity. Skin hydration relieves symptoms and, if a skin toxicity does occur, can reduce exacerbations that may lead to secondary infections [1, 13]. Cleansers should have a pH close to that of skin (~ 5), and basic and neutral pH cleansers should be avoided [1, 3, 13]. Emollients may include ingredients such as shea butter and niacinamide to improve skin barrier; and/or *Aqua Posae Filiformis* (APF) and microresyl to reinforce skin physical barrier and maintain a well-diversified microbiota. Urea can be important, particularly for hands and feet, since it has both exfoliating and hydrating actions [21]. However, urea should be avoided in cases of radiation dermatitis and used with caution in red or irritated areas. Topical moisturizers and emollients help maintain skin hydration, reducing the likelihood of fissures and cracks [3, 13]. Protecting epidermal barrier function can also reduce the risk of xerosis induced by treatments. Although not in the cancer setting, ceramide-rich moisturizers and cleansers have been shown to relieve moderate eczema due to their ability of modulate water and maintain skin permeability [22]. Increasing ceramides can help maintain a healthy diversified microbiome. Finally, gentle cover makeup may help cover lesions and improve quality of life and self-esteem for patients [3].

It is imperative to educate patients to avoid products containing allergens and irritants (preservatives, fragrances, perfumes) [14]. Alkaline cleansing products (especially with pH 7 or higher) should be avoided due to the potential for triggering inflammation and reducing microbiome diversity [14]. Use alpha hydroxy acids (lactic acid, glycolic acid) with caution, since they may alter skin surface pH and/or be irritating [14].

### Active cosmetics used in specific toxicities

#### Xerosis/pruritus

Xerosis and pruritus are associated with many types of cancer treatments (Table 1) and typically appear several weeks after initiation of antineoplastic treatment [3, 13, 23]. Xerosis, which often presents as diffuse fine scaling, may occur in up to 84% of patients, depending on the treatment [12, 13]. When present on hands or feet, xerosis may lead to pain and fissuring [3, 24]. Valentine et al. conducted a systematic review of xerosis in patients receiving targeted therapies, which included meta-analysis of clinical trials and covered the period of 1966 to 2013.The findings revealed that xerosis occurred in 17.9% of cancer-treated patients overall and was high grade in 1.0% [25]. Risk for xerosis was affected by older age, concomitant medications and comorbidities, and pre-existing malignancies or skin problems [25]. If xerosis progresses, asteatotic eczema can occur and can be a site for secondary infection with *Staphylococcus aureus* or *Herpes simplex* [3].

**Table 1:** Summary of adverse effects and management approaches

Type of AE	Type of treatment	Proactive (prevention/step 0)	Reactive (management in case of AE)
Xerosis/pruritus	• Targeted therapy (EGFR inhibitors, MEK inhibitors, FGFR inhibitors, BRAF inhibitors, PI3K inhibitors) • Cytotoxic chemotherapy • Radiation therapy • Immune checkpoint inhibitors	• Initiate at same time as cancer treatment • Gentle cleanser close to skin pH with lipid replenishing ingredient • Moisturizers, including moisturizers able to maintain diverse skin microbiome (emollient “plus”), with key ingredients such as shea butter, niacinamide, and/or ceramides • Apply moisturizer to face, hands, feet, neck, and back daily plus re-apply as needed	• Gentle cleanser close to skin pH with lipid replenishing ingredient • Emollients “plus” balm, able to rebalance microbiome, with key ingredients such as shea butter, niacinamide, and/or ceramides • Preferred formulas: moisturizing cream and balms • Urea 3–10% in case of severe xerosis; avoid in red/irritated areas and with radiation dermatitis • Hydrating lip balms, preferably with sunscreen
			If eczematiform eruption: • TCS/TCI once–twice per day (maximum of 4 weeks for TCS) might be needed if pruritus uncontrolled by dermocosmetics • Antihistamines • GABA agonist • Doxepin/aprepitant/antidepressants • TENS or acupuncture
Maculopapular rash^1^	• Targeted therapy (BRAF inhibitors) • Conventional chemotherapy - Antimetabolites (5-FU etc.) - Alkylating agents (cyclophosamide, cisplatin) - Mitotic inhibitors (taxanes, vinca alkaloids) Immunotherapy (immune checkpoint inhibitors, anti-Nectin 4)	• Emollients “plus” balm, able to rebalance microbiome, with key ingredients such as shea butter, niacinamide, and/or ceramides • Moisturizers with key ingredients such as shea butter, niacinamide, ceramides • Sunscreen with UV broad spectrum UVA/UVB filters to prevent possible PIH in darker phototypes	• Continue Step 0 • Camphor-menthol-hydrocortisone cream • Topical antipruritics
			• TCS (once–twice per day for a maximum of 4 weeks) • Oral antihistamines • Oral corticosteroids for grade 3 toxicities • Oncologist may suspend or alter treatment at grade 3; resume after 2 weeks if rash has faded to 0–1 • Grade 4, admit to hospital
Acneiform rash	• Targeted therapy (EGFR inhibitors including tyrosine kinase inhibitors, MEK inhibitors, mTOR inhibitors) • Multikinase inhibitors	• Gentle cleanser with pH ~ 5 • Moisturizer • Sunscreen with UV broad spectrum (UVA/UVB) on all sun exposed areas. Patients with darker phototypes are at higher risk for PIH	• Continue Step 0 • Moisturizing cream and balms • Barrier repair cream on face such as soothing balm, able to rebalance microbiome with ingredients such as panthenol • Acne dermocosmetics with niacinamide
		• Oral doxycycline^2^ (100 mg BID) or minocycline when using EGFR and MEK inhibitors	• Minocycline/doxycycline • Topical corticosteroids after checking there is no local superinfection • Systemic steroids^3^ • If not enough, oncologist may suspend or alter treatment
Photosensitivity	• BRAF inhibitors • EGFR inhibitors • Multikinase inhibitors • Vandetanib • Chemotherapy (fluorouracil, dacarbazine, methotrexate, paclitaxel, vinblastine) • Radiation therapy	• Gentle cleanser with pH ~ 5 • Sun avoidance: especially direct sun at mid-day* • Sun protection when outside with UV broad spectrum UVB/UVA SPF 50 or greater sunscreens and strong UVA protection, especially with targeted therapy; clothing, sunglasses, and hats; patients with darker phototypes are at higher risk for PIH • Reapply sunscreen q2h • Educate patient that sun can penetrate glass windows and in cloudy weather (home, car, workplace)	• Continue Step 0 • Cool colloidal oatmeal baths may help • Soothing emollients • Aloe vera
			• NSAIDs, other painkillers • Topical corticosteroids
Skin/nail pigmentation changes	• Conventional chemotherapy • Antimetabolites (5-FU etc.) • Alkylating agents (cyclophosamide, cisplatin) • Mitotic inhibitors (taxanes, vinca alkaloids) • Cytotoxic (bleomycin) • EGFR/HER inhibitors • Capecitabine • Multikinase, checkpoint inhibitors • MEK/BRAF/VEGFR inhibitors	• Gentle cleanser with pH ~ 5 • Sun avoidance: especially direct sun at mid-day* • Sun protection when outside with UV broad spectrum UVB/UVA SPF 50 or greater sunscreens and strong UVA protection, especially with targeted therapy; clothing, sunglasses, and hats; patients with darker phototypes are at higher risk for PIH • Reapply sunscreen q2h • Opaque nail lacquers • Avoid heat, humidity	• Continue Step 0 • Dermocosmetics containing niacinamide
			• Topical corticosteroids • Hydroquinone for hyperpigmentation (limited to 6 months of use maximum)
Inflammatory hand and foot syndrome	• Conventional chemotherapy - Liposomal doxorubicin - Mitotic inhibitors (taxanes) • Capecitabine (5-FU)	• Moisturizer with urea 10%^4^ • For doxorubicin and taxane infusions – cooling gloves/socks during infusions including 15 min before and 30 min after^5^	• Continue Step 0 • Moisturizing cream and balms • Monitor for change in severity
		**Optional** • Oral steroids for doxorubicin HFS, celecoxib for capecitabine^6^	• TCS (once-twice per day for a maximum of 4 weeks) including under occlusion • Oral dexamethasone • For capecitabine, on/off regimens can help reduce HFS • Oncologists may reduce, interrupt, or discontinue treatment at higher grades
Hyperkeratotic HFS	• Targeted therapy: RAS-BRAF-MEK-ERK inhibitors, multikinase inhibitors	• Gentle cleanser with pH ~ 5 • Moisturizer with urea 10% (2–3 times per day)^7^ • Remove hyperkeratosis before the start of treatment (pedicure) • Soft/padded footwear, avoid walking barefoot, avoid high heels	• Continue Step 0 • Topical urea based 10–40% emollient (2–3 times per day on hyperkeratotic areas) • Refer to podiatrist
		• Clobetasol^8^	• Lidocaine patches/cream (as needed for pain) • Painkillers, NSAIDs • Potent topical corticosteroids • Oncologist may reduce, interrupt, or discontinue treatment at higher grades
Acute radiation dermatitis	• Radiation therapy, enhanced by concomitant chemotherapy or targeted therapy (especially 5-fluorouracil and EGFR inhibitors) but not necessarily by checkpoint inhibitors	• Cleanser with pH ~ 5 • Daily moisturizer* but not to be applied within 2 h before radiation therapy • Sun protection when outside with UV broad spectrum UVB/UVA SPF 50 or greater sunscreens and strong UVA protection, especially with targeted therapy; clothing, sunglasses, and hats; patients with darker phototypes are at higher risk for PIH • Avoidance of skin irritants • Compression stockings for field cancerization on legs • Aloe vera on Day 1 and throughout the course (apply post-treatment daily) • Cocoa butter prophylaxis daily during radiation therapy and one month beyond for darker skinned patients • Sterile transparent film dressing from Day 1 through one week post radiation therapy (applied continuously)	• Gentle skin care • Zinc oxide for low infectious risk dermatitis (non-immunosuppressed patients) • Silvadene if risk of superinfection or refractory to OTC topicals • Drying solution TID as drying/healing agent • Lidocaine 2% gel for pain • Wound hydrogel pads for inflammatory reactions in regions that can support the pad without adhesive (such as inframammary fold) • Medicated powder for initial moist desquamation, also provides antifungal activity
		• Mid-potency TCS (betamethasone/mometasone, betamethasone is superior) once daily from Day 1 and throughout course	• Mid potency TCS • Monitor for secondary infection (culture and use oral antibiotic as needed) • Antibiotics in case of superinfection • Oncologist may interrupt treatment in case of grade 3 ulceration
Chronic radiation dermatitis	• Radiation therapy, enhanced by concomitant chemotherapy or targeted therapy (especially 5-fluorouracil and EGFR inhibitors) but not necessarily by checkpoint inhibitors	• Proper radiation therapy techniques • Gentle cleanser with pH ~ 5 • Moisturizer • Sun protection	• Gentle skin care • Educate patient about risk of late development of skin cancers in treated areas • Thermal water • Thermal cure
			• Pentoxifylline 400 mg TID to prevent radiation induced fibrosis (in later part of radiation therapy or following development of fibrosis; use for at least 6 months as long as it is still improving the condition) • Vitamin E (use with caution in patients taking anticoagulant or antiplatelet therapy)
Alopecia from chemotherapy	• Conventional chemotherapy (alkylating agents, antimetabolites, mitotic inhibitors)	• Scalp cooling for cytotoxic infused chemotherapy^9^ • Gentle shampoo • Moisturizing cream	• Microblading/semi-permanent tattooing of eyebrows
			• Topical bimatoprost can be considered with eyelash loss
Alopecia from hormonal and targeted therapy	• Aromatase inhibitors • Kinase inhibitors: FGF, BRAF, CDK inhibitors • Sonic hedgehog inhibitors	• Gentle shampoo • Moisturizing cream	• Oral vitamins (consider zinc in combination with spironolactone in zinc-deficient patients)
			• Topical 5% and oral minoxidil • PRP for patients who cannot tolerate minoxidil • Spironolactone
Hypertrichosis and trichomegaly	• EGFR inhibitors	• None	• Hypertrichosis: cosmetic techniques, laser, epilation, threading • Trichomegaly: eyelash clipping, referral to ophthalmologist

*BRAF* BRAF gene, *CDK* cyclin dependent kinase, *EGFR* epidermal growth factor receptor gene, *ERK* extracellular signal-regulated kinase, *FGF* fibroblast growth factor, *FGFR* FGF receptor, *HER* human epidermal growth factor receptor, *MEK* mitogen activated protein kinase, *mTOR* mechanistic target of rapamycin, *PI3K* phosphoinositide 3-kinase, *PRP* platelet rich plasma, *RAS* renin angiotensin system inhibitor, *VEGFR* vascular epithelial growth factor

*AE* adverse event, *BID* twice daily, *5-FU* 5 fluorouracil, *GABA* gamma aminobutyric acid, *HFS* hand and foot syndrome, *NSAIDS* nonsteroidal anti-inflammatory drugs, *OTC* over the counter, *q2h* every two hours, *SPF* sun protection factor, *TCI* topical calcineurin inhibitors, *TCS* topical corticosteroids, *TENS* transcutaneous electrical nerve stimulation, *TID* three times daily, *UV* ultraviolet

^1^Warning: Check that rash is not a cutaneous toxicity that would necessitate drug interruption, viral infection, or relapse of previous skin condition. Avoid oral steroids for maculopapular rash > grade 3 without a dermatology consultation. ^2^Can cause photosensitivity. ^3^ In extreme cases, which occur rarely in clinical practice. ^4^With doxorubicin and antimetabolites, may use higher percentage depending on patient response. ^5^When using doxorubicin and taxanes, Bun et al. Support Care Cancer 2018. ^6^When using capecitabine in colorectal patients. ^7^May use higher percentage depending on patient response. ^8^With multikinase inhibitor regorafenib. ^9^Contraindications: hematologic cancers and cancers of head/neck, those receiving platinum-based therapy due to cold sensitivity

^*^Sun avoidance can depend on the region and individual patient variables

Emollients are a key dermocosmetic to employ for xerosis, particularly those containing urea and niacinamide, as they maintain maximal skin hydration [3, 13, 26]. Emollient plus an acidic (pH 5.5) cleanser have been shown to increase stratum corneum hydration (*P* < 0.001) along with decreasing transepidermal water loss (*P* < 0.03) [27]. A small pilot study reported that prophylactic application of a niacinamide-containing emollient reduced the rate of skin toxicities while maintaining good quality of life [28]. Additionally, moisturizing was effective in managing xerosis in a small (*n* = 30) uncontrolled trial of cancer patients [29]. More recently, a systematic review concluded that moisturizer use reduced the occurrence of severe acute radiation dermatitis and there was weak evidence indicating moisturizer use improved quality of life [30]. Patients should also be educated to avoid harsh soaps and basic pH cleansers, limit shower time and avoid hot water, and frequently apply emollients [1, 3]. Ointments may lead to follicular occlusion and folliculitis, so these should be used with caution [3]. However, some patients may need ointments to achieve sufficient relief particularly when present on hands, feet, and very dry areas, or if fissures have occurred [3].

#### Maculopapular rash

Maculopapular rash is also associated with a variety of anticancer treatments and may be both more common and more severe with combination therapies such as immune checkpoint inhibitors with cytotoxic drugs (Table 1) [31]. Maculopapular rashes may appear as early as within the first 2 weeks of anticancer treatment and may be accompanied by non-cutaneous symptoms such as fever [31]. There is evidence to show that Grade 1 rash may be managed with emollients and appropriate skin cleansers [32]. Rash severity may be a surrogate marker of anticancer therapy efficacy, so every effort should be made to manage the patient's symptoms with dermocosmetics and skin care to allow continuation of therapy as long as possible [13].

It is important to verify whether severe rash (grades 3 or higher by the Common Terminology Criteria for Adverse Events definition) is not a cutaneous toxicity that would necessitate drug interruption. In some cases, a topical corticosteroid and antihistamines can be added to allow therapy to continue.

#### Folliculitis (formerly known as acneiform rash)

As shown in Table 1, targeted therapies and multikinase inhibitors are associated with a folliculitis which mimics acne (hence the widespread use of the term acneiform rash) [33]. Folliculitis has drawn attention to cancer therapy associated skin toxicities and is common among patients treated with EGFR inhibitors (up to 100%) [4, 33]. This skin toxicity may be associated with favorable response to anticancer therapy, yet folliculitis can impact patient quality of life and can be of sufficient severity to limit anticancer treatment [2, 33, 34]. Folliculitis usually occurs within 2–4 weeks after initiation of therapy and manifests as erythema, papules, and pustules; unlike acne, folliculitis does not include comedonal lesions and may cause itchiness, pain, and spontaneous lesional bleeding [4, 33]. The distribution of lesions is similar to that of acne (face, trunk), but may also include lower trunk, arms/legs, and buttocks (these areas are often a sign of superinfection) [2, 33]. Cyclines at low doses with anti-inflammatory activity should be included in prevention; however, they may be associated with only mild efficacy as treatment [4, 33, 34]. Topical dapsone gel prophylaxis may also have utility [4]. Moisturizers used twice daily are recommended, particularly those with urea 5% to 10%, along with photoprotection [34].

Topical retinoids, benzoyl peroxide (BPO), and azelaic acid should be avoided due to potential for burning/irritation, although some authors use BPO at low concentration in the evening [3]. Dermocosmetics with niacinamide can be recommended as proactive/reactive approaches. We believe that it would be helpful to have a study showing efficacy of dermocosmetics as prophylaxis/treatment in this setting. Generally, patients should avoid sun exposure and frequent washing with hot water [34]. Bacterial culture should be considered in the case of suboptimal response, particularly when infection is suspected [2, 34].

#### Photosensitivity

Sun exposure and ultraviolet (UV) light can exacerbate skin toxicities, including rash and xerosis [3]. UVA has a major role in this setting, and it is important to educate patients about the need to use broad-spectrum sunscreen with UVA (covering long UVA wavelengths: 330–450 nm) and UVB filters along with good sun protection (clothing, hats, UV films on windows) [1, 35]. In addition, sun exposure can lead to pigmentation changes in dark-skinned patients [3]. If the patient finds sunscreen irritating, sunscreens with physical blocks such as zinc oxide or titanium dioxide can be used [1].

#### Skin/nail changes

Anticancer treatments can cause nail changes by affecting the rapidly dividing matrix cells of the nail plate [36]. Nail changes are common with cytotoxic therapy, EGFR inhibitors, and radiotherapy (Table 1); they are usually—but not always—transient and resolve with discontinuation of therapy [2, 36]. They often occur after 1–2 months of treatment and can include onycholysis, paronychia, and changes in pigmentation/texture [2]. While some nail changes have primarily a cosmetic aspect, others may cause significant pain [36]. This toxicity has a high psychological impact and can be difficult to treat [36]. Patients should be educated about the potential for nail changes, including a discussion about preventive measures such as minimizing pressure, trauma, and friction on the nails [36]. Irritants such as frequent or prolonged water exposure, aggressive manicures, nail biting, and artificial nails should be avoided during treatment. Generally, nail toxicities are managed conservatively and do not require drug withdrawal [1]. However, discontinuation may be needed in severe cases [36]. Application of a nail polish to protect the nails as well as liquid bandages have been used to prevent nails from breaking [1, 3].

#### Inflammatory hand and foot syndrome

Hand and foot syndrome (HFS) typically presents as an inflammatory condition, but multikinase inhibitors induce a hyperkeratotic HFS which differs from chemotherapy-induced HFS in pathophysiology, symptoms, and treatment options (discussed below) [37]. HFS with cytotoxic therapies is characterized by dysesthesia, erythema, and scaling and often has a symmetrical, diffuse presentation [38]. Patients may also have vesicles and bullae, which can desquamate [2]. Early recognition of HFS is essential, since this toxicity can quickly progress to debilitating stages [3].

Several preventive approaches have been studied—including use of COX inhibitors, pyridoxine, and urea creams [34, 37, 39]. According to a recent meta-analyses of randomized controlled studies, celecoxib and urea cream were associated with significant risk reduction and/or reduction of severity [37, 39]. Notably, celecoxib reduced risk of HFS in patients receiving capecitabine, urea cream was somewhat less effective in capecitabine-treated patients but showed greater risk reduction in sorafenib-treated patients [37]. Salicylic or urea ointments may also be beneficial [13]. Educate patients at risk to avoid irritants to hands and feet (environmental, chemical, other) [34]. Table 1 presents recommendations from our clinical experience.

#### Hyperkeratotic HFS (under multikinase inhibitors)

A high proportion (up to 71%) of patients treated with multikinase inhibitors develop HFS; the incidence varies both with tumor type and the specific multikinase inhibitor [34, 38, 40]. Multikinase inhibitor HFS is sometimes referred to as hand-foot reaction, to distinguish from the HFS associated with conventional agents such as capecitabine [38]. The onset of HFS with multikinase therapy may be more rapid than with cytotoxic therapy: within days to a few weeks compared to weeks/months after treatment initiation [38]. Unlike the clinical presentation of inflammatory HFS, multikinase HFS often manifests with dysesthesia and erythema along with pain, fissures, blisters and hyperkeratosis; erythematous plaques may also develop [2, 38, 41]. It is typically localized to areas of hands and feet that serve as pressure points [38, 41]. In this setting, salicylic acid or urea may be useful, and the higher urea concentrations may be needed: 10% for prevention up to 40% for management of hyperkeratotic HFS [4]. Patients should be educated to avoid irritants to hands and feet (environmental, chemical, mechanical), long walks, and walking with bare feet [1]. Table 1 presents recommendations from our clinical experience.

#### Acute radiation dermatitis

Among the side effects of radiotherapy, radiation dermatitis—or radiodermatitis—is among the most common [3, 42]. It can have a range of clinical manifestations from erythema or discomfort to severe confluent moist desquamation [3]. Up to 90% of patients with cancer develop acute skin toxicities during radiation therapy [43, 44]. Skin damage occurs in the area treated with radiation and can be exacerbated by systemic therapies [45]. Use of radiation therapy in combination with systemic therapy can worsen skin reactions, causing severe xerosis, skin thinning, and even necrosis of the upper skin layers [3]. It has been theorized that the makeup of the human microbiome is associated with the severity of radiation dermatitis. Skin microbiome analysis and appropriate management with personal care products to balance the microbiome could be a tool in the future to prevent or decrease the severity of radiodermatitis symptoms.

Acute radiation dermatitis occurs within 1 to 4 weeks of treatment and may lead to dose reduction or discontinuation of anticancer therapy [3, 42]. “Chronic radiation dermatitis” is usually defined as effects occurring after radiation therapy has completed (see below) [42].

Proactive skin care can safely minimize skin reactions and help support epidermal barrier function for patients undergoing radiotherapy [43, 46]. Berger et al. reported that a kit of 5 commercially available skin care products was associated with > 92% good–excellent tolerability of irradiation, and heavy product users had less skin toxicities vs low users [43]. Sun protection is also important, depending on location, since radiation therapy can increase sensitivity to UV light [42]. Topical treatments with hyaluronic acid may be beneficial, and those with niacinamide, panthenol, glycerin, allantoin, or squalene may be soothing [42]. In breast cancer patients, a skincare regimen including thermal water (La Roche-Posay), cleansers, moisturizer, healing balm, and sunscreen was associated with significantly less (*P* ≤ 0.0001) severe radiation dermatitis vs none or infrequent use of the skincare regimen [47].

Along with recommendations in Table 1, supportive therapy should be utilized as needed [42]. These may include the following: pain management, wound healing, psychological support, cosmetic interventions, special dressings for wounds with alginate, Mepilex® dressing with skin openings in axilla area, Mepitel Film® at beginning of treatment, silver, hydrocolloids, hydrofilm, and photobiomodulation therapy (LEDs) [42]. If hyperpigmentation occurs, hydroquinone or tretinoin may be considered, but should be initiated no earlier than 2 months after radiation therapy; cocoa butter may be useful in patients with darker skin types. Interruption of anticancer therapy is at the discretion of the oncology team and will depend on the disease type. For head and neck tumors, an attempt should be made to continue therapy until Grade III symptoms occur unless the course of radiotherapy is nearly complete. For some other disease types, short breaks are not overly concerning [48].

#### Chronic radiation dermatitis

Chronic radiation dermatitis can present as persistent pigmentary alterations (ranging from mild to severe), atrophy, skin necrosis, and telangiectasias [1]. Moisturizer and sunscreen have utility as preventive options, and thermal water can be used if symptoms occur (Table 1). In chronic radiation dermatitis, pentoxifylline + vitamin E to prevent radiation -induced fibrosis can be both prophylactic and reactively used. This is used mostly in breast cancer and should be prescribed by a clinician. Patients should be educated about their elevated risk for skin cancer in the long term. Supportive therapy for chronic radiation dermatitis includes pain management, wound healing, psychological support, cosmetic interventions, and physiotherapy for fibrosis and sclerosis. Some clinicians have hesitated to use deodorant and shampoo during treatment, but there is no evidence supporting a detrimental effect, and these may be allowed for patients [49].

### Alopecia

Alopecia can occur from chemotherapy, targeted therapy, or hormonal therapy and, depending on the anticancer treatment, may have distinct clinical management strategies. In all cases, use of hats, scarves, and wigs can be helpful, along with psychological support and cosmetic interventions. When accompanying chemotherapy, it is important to check levels of thyroid stimulating hormone, vitamin D, zinc, and ferritin and correct deficiencies as needed. Targeted therapies that can induce alopecia include multikinase inhibitors, BRAF inhibitors, FGF inhibitors, hedgehog inhibitors, and CD4 inhibitors. Management of targeted-therapy induced alopecia can be difficult, and scalp cooling can be used in some chemotherapy-induced cases of alopecia but should not be used in association with endocrine therapies. For alopecia associated with hormonal therapy, minoxidil may be prescribed and platelet rich plasma for those who cannot tolerate minoxidil [50, 51]. Finasteride and dutasteride should be avoided in female breast cancer patients [52].

#### Hypertrichosis and trichomegaly

Hypertrichosis can be associated with paraneoplastic syndromes, and it may be difficult to determine whether it is an adverse event related to treatment or syndrome-related. Before treating as an AE of cancer treatment, non-linkage to a paraneoplastic syndrome must be verified. Trichomegaly, manifesting as thickened, elongated, and curled lashes and brows, can occur in patients treated with EGFR inhibitors [2]. This may be managed by eyelash trimming or laser removal [2]. Psychological support and cosmetic interventions may also be useful. Epilation creams with glycolic acid and waxing should be avoided.

### Keratinocytic hyperproliferative tumors under BRAF-I

Approximately 20% of patients treated with BRAF-inhibitor monotherapy will develop secondary skin tumors and other hyperproliferative lesions, such as squamous cell carcinoma (SCC) and keratoacanthomas (KA) [4]. However, metastatic spread has not been reported from those secondary cancers, and there is usually no need for dose modification or treatment interruption. Interestingly, concomitant treatment with MEK inhibitors decreases the incidence of skin toxicities compared with a BRAF inhibitor alone by blocking the MAP kinase pathway downstream. As BRAF inhibitors are phototoxic, photoprotection with broad spectrum UVB and UVA protection is recommended [4].

## Discussion

While there is increasing appreciation of the widespread nature of skin toxicities with anticancer treatment, many persons involved in cancer care relegate discussions about dermatologic adverse events to a secondary priority [11, 14]. In addition to often being a visible reminder of cancer, skin toxicities can also cause significant emotional and physical discomfort [1, 11]. Treatment interruptions occur in as many as 50% of cancer treated patients—depending on the type of treatment and the severity of the side effect [1]. In many areas, access to dermatologists can be difficult in a timely fashion [11]. Yet the advances in cancer treatments mean that there will be an expanding population of cancer patients with skin toxicities [14]. Dermatologists have a vital role in working with the cancer care team on how to manage skin toxicities with a goal of optimizing outcomes [1, 11]. Creation of proactive and preemptive strategies to mitigate skin toxicities can be an effective way to minimize the likelihood and severity of skin problems for cancer patients [1, 14]. Daily skin care routines should be used for prevention and treatment. They should be discussed with the patient, to help them avoid skin-care products that could exacerbate problems. Active cosmetics can be used to manage skin toxicities of different types; these products have good efficacy but better tolerance and may allow use of alternative treatments or dosages. Severity of a skin toxicity should be assessed by checking for lesions affecting 10% or more skin surface area, fever, pain, involvement of the mucosa, and blood abnormalities [14]. A photographic glossary with key features identifying specific skin toxicities may help support prompt and effective management [14].

In addition to the evidence discussed in the introduction about how dermatology involvement can reduce skin toxicities and improve patient outcomes, there are also emerging data about use of dermocosmetics in this setting, as illustrated in the following examples. Prophylactic use of a niacinamide-based emollient maintained quality of life in women being treated for breast cancer (*n* = 73) [26]. In addition, preemptive skin care reduced the incidence of severe skin toxicities by 50% vs reactive skincare [53]. Luftner et al. studied use of a supportive and barrier-protective skincare regimen (a kit including 12 products) in prevention and treatment of skin toxicities during chemotherapy (*n* = 147) [54]. Again, skin toxicities were reported more frequently in casual users of the regimen compared to those who used it daily (*p* = 0.029) [54]. Erythema and desquamation were also more common in those who used skincare less frequently (*p* < 0.05) [54]. In addition, a mobile application (app) for daily use of skincare was associated with a trend toward reduced cases of radiation dermatitis above grade 2 [55]. Daily use of an emollient containing an extract of *Aquaphilus dolomiae* reduced xerosis severity and improved quality of life score in an observational study of 319 cancer patients with xerosis [56].

Skin care has an important supportive role for patients with cancer [13]. Skin toxicities are primarily associated with skin barrier dysfunction and dysbiosis, and maintaining good skin barrier function can reduce the occurrence and severity of symptoms [13]. The literature is clear that use of emollients and mild soaps contributes to improved skin physiology and appearance [13]. Non-occlusive emollients are a well-established approach to treat maculopapular skin rash, one of the most common of skin toxicities [13]. Since exposure to sunlight can worsen rashes, photoprotection is a prudent addition, depending on time of year, likelihood of patient exposure, and geographic location [57]. It is important to begin skin care at the initiation of anticancer treatment [13]. Patients should be encouraged to report symptoms throughout therapy, and additional topical or systemic treatments implemented according to existing guidelines [13]. We hope the recommendations in this publication will enhance creation of skin care protocols to optimize therapeutic outcomes.

## Data Availability

Not applicable.

## Declarations

### Disclaimer

MASCC does not endorse any brand names of therapeutics identified in this document or in any other documents.
